# Gastrodin alleviates cardiomyocyte senescence by regulating autophagy through the AMPK/mTOR/4EBP1 pathway

**DOI:** 10.3389/fphar.2026.1851573

**Published:** 2026-06-16

**Authors:** Mingyue Yao, Simin Zhang, Yan Wu, Yunqian Huang, Yao Song, Lei Wang, Shan Li, Longhao Jia, Baihui Yao, Bo Li, Li Dong

**Affiliations:** 1 The Affiliated Traditional Chinese Medicine Hospital, Southwest Medical University, Luzhou, China; 2 The Key Laboratory of Integrated Traditional Chinese and Western Medicine for Prevention and Treatment of Digestive System Diseases of Luzhou City, The Affiliated Traditional Chinese Medicine Hospital, Southwest Medical University, Luzhou, China

**Keywords:** AMPK/mTOR/4EBP1 pathway, autophagy, cardiomyocytes, gastrodin (GAS), senescence

## Abstract

**Objective:**

To investigate whether Gastrodin (Gas) attenuates doxorubicin (Dox)-induced cardiomyocyte senescence and apoptosis by regulating the AMPK/mTOR/4EBP1 signaling pathway and autophagy-related processes.

**Methods:**

A Dox-induced senescence model was established in H9c2 cardiomyocytes, and different concentrations of Gas (0.5 mM and 1 mM) were used for intervention. Senescence phenotypes were evaluated using SA-β-gal staining and senescence-associated proteins (P16, P21, and P53). Furthermore, the autophagy inhibitor 3-MA and the AMPK inhibitor Compound C (CC) were applied. Combined with Western blotting, Annexin V/PI flow cytometry, and immunofluorescence techniques, the expression levels of senescence-related proteins, autophagy-related markers (LC3-II/I, Beclin-1, and p62), and the phosphorylation levels of key proteins in the AMPK/mTOR/4EBP1 signaling pathway were analyzed to explore the potential mechanism of Gas.

**Results:**

Compared with the Dox model group, Gas intervention dose-dependently reduced the percentage of SA-β-gal-positive cells, downregulated the protein expression levels of P16, P21, P53, and γ-H2AX, and significantly inhibited apoptosis. Mechanistically, Gas treatment partially ameliorated the abnormalities in autophagy-related markers induced by Dox, as evidenced by increased expression of LC3 and Beclin-1 and decreased accumulation of p62, and this ameliorative effect was partially attenuated by 3-MA. Further signaling pathway analysis demonstrated that Gas significantly increased the p-AMPK/AMPK ratio while decreasing the p-mTOR/mTOR and p-4EBP1/4EBP1 ratios. After AMPK inhibition with CC, the activation effect of Gas on autophagy and its protective effects against senescence and apoptosis were both attenuated.

**Conclusion:**

These findings indicate that Gas alleviates Dox-induced cardiomyocyte senescence and apoptosis, and its protective effects may be associated with regulation of the AMPK/mTOR/4EBP1 signaling pathway and improvement of autophagy-related processes.

## 1 Introduction

Cardiovascular diseases remain the leading cause of death worldwide. According to the World Health Organization, approximately 19.8 million people died from cardiovascular diseases globally in 2022, accounting for about 32% of all deaths worldwide (World Health, 2025). With the aggravation of population aging, cardiac aging has become an important biological basis of elderly cardiovascular events such as heart failure. The aging heart undergoes degenerative changes in both structure and function, including cardiomyocyte senescence, decreased vascular compliance, and chronic low-grade inflammation (Ungvari et al., 2018). Current clinical management primarily focuses on risk factor control and complication management. Although these strategies can improve prognosis, reversible intervention strategies targeting the mechanisms of cardiac aging itself remain relatively limited.

Cellular senescence is one of the core mechanisms driving cardiac aging. Although cardiomyocytes are terminally differentiated cells (Ahuja et al., 2007), they can still exhibit senescence-like phenotypes under aging or pathological stress conditions. These phenotypes are characterized by increased SA-β-gal activity and upregulated expression of senescence-associated proteins, including P16, P21, and P53. These alterations collectively disrupt the myocardial microenvironment and promote cardiac functional decline and the progression of heart failure (Tang et al., 2020; Redgrave et al., 2023; Zhai and Sadoshima, 2024). Autophagy, as a key process for maintaining intracellular homeostasis, generally shows a declining trend during cardiovascular aging. Age-related impairment of autophagy occurs concomitantly with cardiovascular structural remodeling and functional deterioration (Shirakabe et al., 2016; Abdellatif et al., 2018), suggesting that restoring autophagic homeostasis may represent an important mechanistic entry point for intervening in cardiomyocyte and cardiac aging phenotypes.

Gastrodin (Gas) is the principal active component of *Gastrodia elata* and has demonstrated antioxidant, anti-inflammatory, and anti-aging effects in multiple aging-related models. Studies have shown that Gas can extend the lifespan of *Drosophila*, enhance antioxidant enzyme activities, and improve resistance to oxidative stress, suggesting its potential role in delaying aging (He et al., 2021). In addition, Gas has exhibited protective effects in aging-related disease models through mechanisms such as regulating mitochondrial function (Zhang Y. et al., 2023), inhibiting inflammatory pathways (Yin et al., 2024), and ameliorating neurodegenerative changes (Li J. et al., 2025). Our previous study found that Gas showed a tendency to extend lifespan in *Caenorhabditis elegans* (Li B. et al., 2025). Based on this finding, further preliminary experimental results demonstrated that Gas significantly ameliorated abnormalities in autophagy-related markers in animal models and promoted the upregulation of AMPK phosphorylation levels, suggesting that it may participate in the regulation of aging-related processes through modulation of the AMPK-centered energy-sensing signaling pathway. Based on these findings, we hypothesized that Gas exerts protective effects against Dox-induced cardiomyocyte senescence by activating AMPK, inhibiting the downstream mTOR/4EBP1 signaling pathway, and thereby regulating autophagy-related processes. To test this hypothesis, we used a Dox-induced H9c2 cardiomyocyte senescence model to systematically evaluate the effects of Gas on senescence phenotypes, apoptosis, autophagic activity, and the AMPK/mTOR/4EBP1 pathway. Pharmacological inhibitors were further employed for functional validation, aiming to provide new experimental evidence and mechanistic insights for natural product-based interventions targeting myocardial aging.

## 2 Materials

### 2.1 Experimental cells

The rat H9c2 cardiomyocyte cell line was purchased from procell life science & technology co., ltd (Batch No. CL-0089) and was used for subsequent experiments after three passages.

### 2.2 Drugs and reagents

Doxorubicin hydrochloride (Doxorubicin hydrochloride, MCE, China, HY-15142), gastrodin (Gastrodin, MCE, China, HY-N0115), 3-methyladenine (3-Methyladenine, MCE, China, HY-19312), Compound C (Dorsomorphin dihydrochloride, MCE, China, HY-13418), dimethyl sulfoxide (DMSO, MCE, China, HY-Y0320C), high-glucose DMEM medium (Procell, China, PM150210), penicillin–streptomycin solution (100×) (Beyotime, China, C0222), fetal bovine serum (FBS, PAN), trypsin digestion solution (0.25% trypsin) (Beyotime, China, C0201), 1× PBS buffer (pH 7.2–7.4) (Solarbio, China, P1020), senescence β-galactosidase staining kit (Beyotime, China, C0602), Triton X-100 (Solarbio, China, IT9100), bovine serum albumin BSA-V (Solarbio, China, A8020), AF488-conjugated goat anti-rabbit IgG (Beyotime, China, A0423), skim milk powder (Solarbio, China, LP0033B), serum-free cell freezing medium (New Cell & Molecular Biotech, China, C40100), SDS-PAGE protein loading buffer (5×) (Beyotime, China, P0015L), 20× TBST buffer (Solarbio, China, T1082), Omni-Easy™ one-step stain-free PAGE gel rapid preparation kit (Yeasen, China, PG221, PG222, PG223), P53 (Proteintech, China, 10442-1-AP), P21 (Proteintech, China, 28248-1-AP), P16 (Proteintech, China, 28416-1-AP), LC3-II/I (Affinity, China, AF5402), p62 (Affinity, China, AF5384), Beclin-1 (Affinity, China, AF5128), Phospho-AMPK (Affinity, China, AF3423), AMPK (Affinity, China, AF6423), Phospho-mTOR (Affinity, China, AF3308), mTOR (Affinity, China, AF6308), Phospho-4EBP1 (Affinity, China, AF3830), and 4EBP1 (Affinity, China, AF6432).

### 2.3 Experimental instruments

Chemiluminescence imaging system (Qinxiang, China, Chemiscope 6200), vertical electrophoresis system (Bio-Rad, China, Mini-PROTEAN), fluorescence microscope (EVOS, United States, FL Auto2), inverted fluorescence microscope (Nikon, Japan, ECLIPSE Ti2-U), and flow cytometer (BD, United States, FACSCanto II).

## 3 Methods

### 3.1 Cell culture

Rat H9c2 cardiomyocytes were retrieved from liquid nitrogen storage and rapidly thawed in a 37 °C water bath, followed by immediate transfer into complete culture medium (high-glucose DMEM supplemented with 10% fetal bovine serum and 1% penicillin–streptomycin). The cell suspension was centrifuged at 67 ×g (r = 6 cm) for 3 min at room temperature. After discarding the supernatant, cells were resuspended in fresh complete medium and seeded into 10 cm culture dishes. Cells were cultured in a humidified incubator at 37 °C with 5% CO_2_. After stable growth was achieved, cells were passaged, and at least three passages were performed before experimentation. Subsequent treatments were carried out when the cell confluence reached approximately 80%.

### 3.2 Cell grouping

To evaluate the effects of Gas on Dox-induced H9c2 cell senescence and its potential mechanisms, three independent experiments were conducted, including dose screening, autophagy inhibition, and AMPK inhibition experiments. When cell confluence reached approximately 80%, cells were treated with Dox (0.1 μM) for 24 h to induce a senescence-like phenotype (Foglio et al., 2023). Subsequently, based on preliminary experimental results, Gas (0.5 mM or 1 mM) was added according to the grouping design and incubation continued for an additional 24 h. The Control group received an equal volume of vehicle throughout the experiment. After treatment, cells were collected for SA-β-gal staining, immunofluorescence, Western blotting, and flow cytometry analysis.

#### 3.2.1 Dose screening experiment

Control group (Control), model group (Dox), low-dose group (Dox + 0.5 mM Gas), and high-dose group (Dox + 1 mM Gas).

#### 3.2.2 Autophagy inhibition experiment

Control group (Control), model group (Dox), treatment group (Dox + 1 mM Gas), autophagy inhibitor group (Dox + 3-MA), and treatment plus autophagy inhibitor group (Dox + Gas + 3-MA). In this experiment, 3-MA (5 mM) was added simultaneously during the Dox treatment stage and maintained throughout the Gas intervention stage.

#### 3.2.3 AMPK inhibition experiment

Control group (Control), model group (Dox), treatment group (Dox + 1 mM Gas), pathway inhibitor group (Dox + CC), and treatment plus pathway inhibitor group (Dox + Gas + CC). Compound C (CC, 10 μM) was added during the second stage (Gas intervention stage). In the treatment plus pathway inhibitor group (Dox + Gas + CC), Gas and CC were administered simultaneously during the 24 h Gas intervention period.

### 3.3 SA-β-gal staining

Cells were seeded into 6-well plates. After treatment, the culture medium was discarded, and cells were washed once with PBS. Then, 1 mL β-galactosidase fixation solution was added to each well, and cells were fixed at room temperature for 15 min. After washing three times with PBS (3 min each time), 1 mL staining working solution was added to each well. The edges of the plate were sealed with sealing film, and the plate was incubated overnight at 37 °C in a CO_2_-free incubator protected from light. Before observation, the staining solution was discarded and replaced with PBS. Images were captured under an inverted bright-field microscope, and the percentage of SA-β-gal-positive cells was calculated.

### 3.4 Immunofluorescence staining

Cells were seeded into 24-well plates. After treatment, the medium was discarded and cells were washed three times with PBS. Cells were fixed with 4% paraformaldehyde at room temperature for 10 min and permeabilized with 0.5% Triton X-100 at room temperature for 10 min. After PBS washing, cells were blocked with 5% BSA at room temperature for 30 min. Primary antibodies diluted in 5% BSA were added, and the plate edges were sealed with sealing film, followed by incubation overnight at 4 °C. The next day, cells were washed with PBS and incubated with fluorescence-conjugated secondary antibodies at room temperature in the dark for 1 h. Nuclei were stained with DAPI (1:1000) at room temperature for 1 min. After PBS washing, antifade mounting medium was added, and images were observed and captured using an EVOS fluorescence microscope.

### 3.5 Western blot

After completion of each experiment, cells were collected and lysed on ice with RIPA lysis buffer containing 1 mmol/L PMSF for 15–20 min, followed by centrifugation at 9,660 × g (r = 6 cm) at 4 °C for 15 min. The supernatant was collected. Protein concentrations were determined using the BCA method and normalized to 4 μg/μL. A 5× loading buffer was added at a ratio of 4:1, followed by denaturation at 100 °C for 10 min. A total of 10 μg protein per sample was loaded and separated by 10% SDS-PAGE electrophoresis (80 V for 30 min, then 120 V for 60 min). Proteins were transferred to PVDF membranes at 100 V constant current for 90 min. After blocking with 5% skim milk at room temperature for 1 h (5% BSA was used for phosphorylated proteins), membranes were incubated with primary antibodies overnight at 4 °C. Membranes were washed three times with TBST and incubated with HRP-conjugated secondary antibodies (1:5000) at room temperature for 1 h. After visualization with ECL reagent, protein bands were captured using a chemiluminescence imaging system, and grayscale values were quantified using ImageJ software.

### 3.6 Flow cytometry

Cells were seeded into 6-well plates. After treatment, the culture medium was discarded, and cells were washed once with PBS. Then, 0.5 mL trypsin was added to digest cells for approximately 1 min 30 s. Subsequently, 1 mL complete medium was added to terminate digestion, and the cell suspension was collected. Cells were centrifuged at 151 ×g (r = 6 cm) for 5 min at room temperature. After resuspension in PBS, centrifugation was repeated and the supernatant was discarded. Cells were gently resuspended in 195 μL Annexin V-FITC binding buffer, followed by sequential addition of 5 μL Annexin V-FITC and 10 μL propidium iodide (PI) staining solution. After gentle mixing, cells were incubated at room temperature in the dark for 10–20 min and immediately analyzed by flow cytometry to determine the proportions of early and late apoptotic cells.

### 3.7 Statistical analysis

Statistical analysis was performed using GraphPad Prism 10.0 software. Experimental data were expressed as mean ± standard deviation ( $\bar{x}$ ±s). Comparisons between two groups were performed using an unpaired t-test, while comparisons among multiple groups were conducted using one-way analysis of variance (one-way ANOVA). Multiple comparisons between groups were performed using the LSD-t test. A value of P < 0.05 was considered statistically significant.

## 4 Results

### 4.1 Gastrodin inhibits Dox-induced cellular senescence in a dose-dependent manner

**FIGURE 1:**
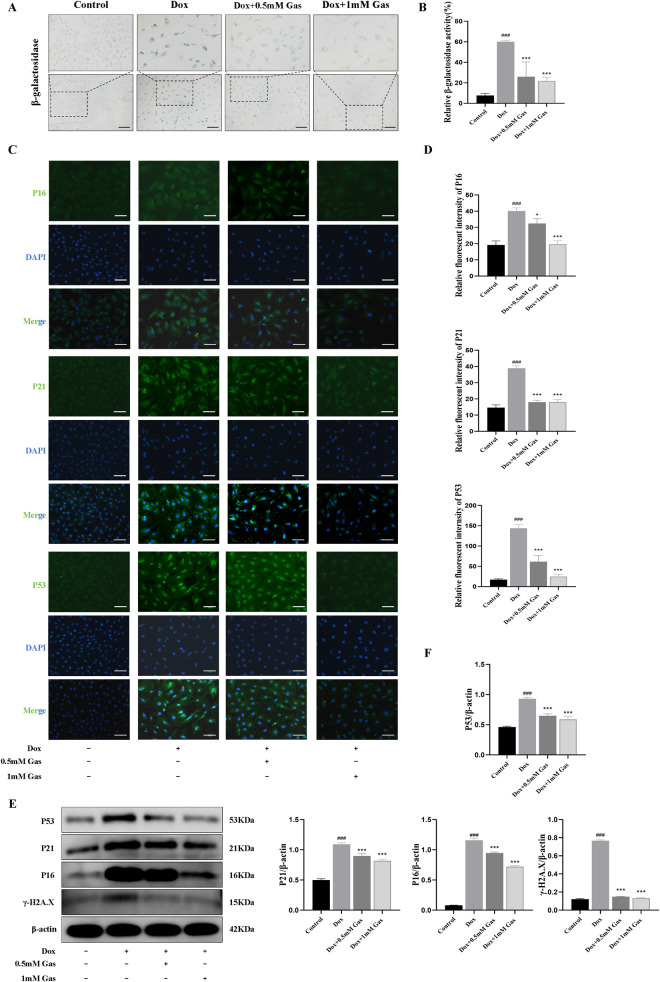
Effects of gastrodin on SA-β-gal staining and senescence-associated proteins. **(A)** Representative images of SA-β-gal staining **(B)**. Quantification of SA-β-gal-positive cell percentage **(C)**. Immunofluorescence detection of P16, P21, and P53 expression **(D)**. Quantitative analysis of immunofluorescence intensity of P16, P21, and P53; **(E)**. Western blot analysis of P16, P21, P53, and γ-H2AX expression levels; **(F)**. Quantification of grayscale values of P16, P21, P53, and γ-H2AX protein bands. Compared with the Control group: ###p < 0.001; compared with the Model group: *p < 0.05, ***p < 0.001; magnification ×10, scale bar = 200 μm.

To clarify the effect of gastrodin (Gas) on doxorubicin (Dox)-induced cellular senescence, the effects of different concentrations of Gas on senescence phenotypes and related markers were first evaluated. SA-β-gal staining results (Figures 1A,B) showed that, compared with the Control group, Dox treatment markedly increased the formation of senescence-associated blue precipitates. After treatment with 0.5 mM and 1 mM Gas, the blue precipitates were significantly reduced, with a more pronounced effect observed at 1 mM Gas (p < 0.05).

At the molecular level, immunofluorescence staining (Figures 1C,D) and Western blot analysis (Figures 1E,F) were performed to detect the expression of key senescence marker proteins P16, P21, and P53. Dox stimulation significantly increased both the fluorescence intensity and protein expression levels of these three proteins (p < 0.05). Following Gas treatment, their expression levels decreased in a dose-dependent manner (p < 0.05), with the 1 mM Gas group showing a more significant reduction. In addition, Western blot analysis (Figures 1E,F) showed that the expression of the DNA damage-associated senescence marker γ-H2AX was significantly increased following Dox treatment, whereas Gas intervention markedly reduced its expression level (p < 0.05). These results indicate that Gas effectively alleviates Dox-induced senescence in H9c2 cells in a dose-dependent manner, as evidenced by a reduced proportion of senescent cells and downregulation of the core senescence markers P16, P21, P53, and γ-H2AX.

### 4.2 The autophagy inhibitor 3-MA reverses the anti-senescence and anti-apoptotic effects of gastrodin

**FIGURE 2:**
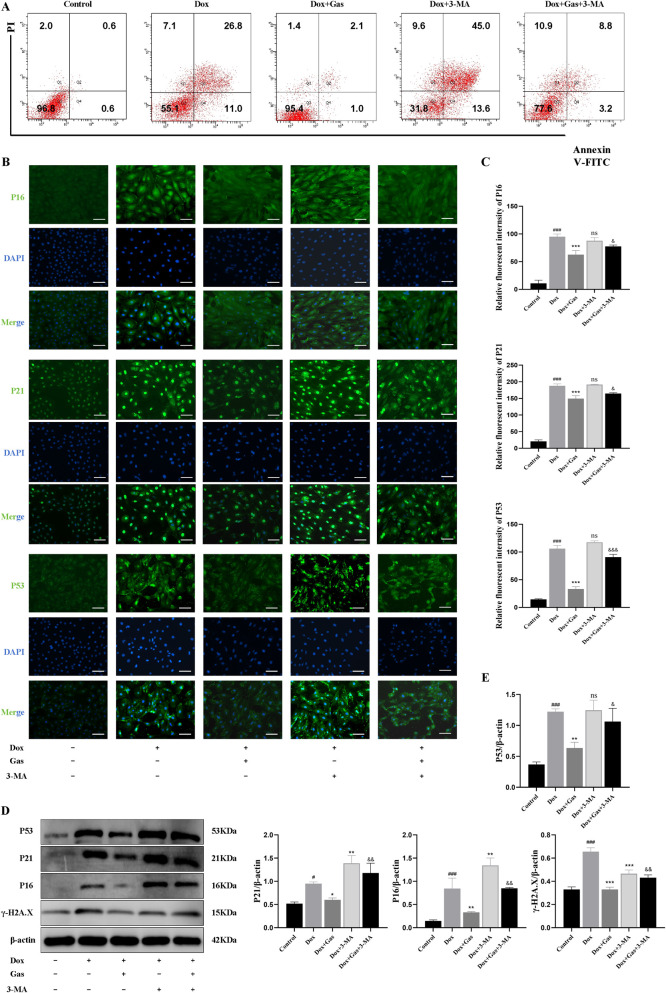
Effects of the autophagy inhibitor 3-MA on the protective effects of gastrodin against senescence-related indicators. **(A)** Apoptosis analysis by flow cytometry **(B)**. Immunofluorescence staining for detection of P16, P21, P53, and γ-H2AX expression **(C)**. Quantitative analysis of immunofluorescence intensity **(D)**. Western blot analysis of P16, P21, P53, and γ-H2AX expression **(E)**. Quantitative analysis of P16, P21, P53, and γ-H2AX protein expression levels and corresponding grayscale values. Compared with the Control group: #p < 0.05, ###p < 0.001; compared with the Model group: *p < 0.05, **p < 0.01, ***p < 0.001; compared with the Gas group: &p < 0.05, &&p < 0.01, &&&p < 0.001; ns, no statistically significant difference, p > 0.05; magnification ×10, scale bar = 200 μm.

To investigate whether autophagy is involved in the protective mechanism of Gas, the autophagy inhibitor 3-MA was further applied. Flow cytometry results (Figure 2A) showed that, compared with the Control group, the apoptosis rate in the Model group was significantly increased. Gas treatment markedly reduced the apoptosis rate, whereas compared with the Gas group, the apoptosis rate in the Gas + 3-MA co-treatment group was significantly elevated, indicating that 3-MA partially blocked the anti-apoptotic effect of Gas.

Consistently, immunofluorescence staining (Figures 2B,C) and Western blot results (Figures 2D,E) demonstrated that when Gas was co-administered with 3-MA, the downregulation of P16, P21, and P53 induced by Gas was significantly reversed (p < 0.05). In addition, Western blot analysis showed that the expression of the DNA damage-related senescence marker γ-H2AX was significantly increased in the Gas + 3-MA group compared with the Gas group (p < 0.05). These findings indicate that Gas alleviates Dox-induced cellular apoptosis and downregulates the expression of senescence-associated proteins, whereas the autophagy inhibitor 3-MA partially attenuates these protective effects, suggesting that autophagy-related processes may participate in the anti-senescence and anti-apoptotic effects mediated by Gas.

### 4.3 The autophagy inhibitor 3-MA attenuates the regulatory effects of gastrodin on autophagy-related proteins

**FIGURE 3:**
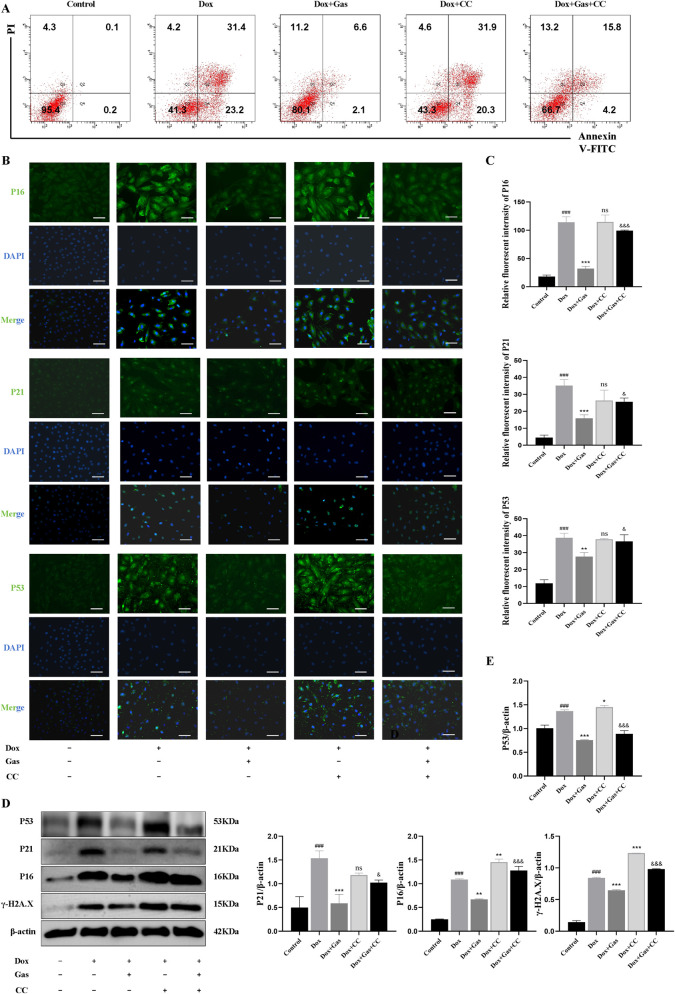
Effects of the autophagy inhibitor 3-MA on gastrodin-mediated regulation of autophagy. **(A)** Immunofluorescence staining for detection of LC3 expression **(B)**. Western blot analysis of P62, Beclin-1, and LC3-II/I expression **(C)**. Quantitative analysis of P62, Beclin-1, and LC3-II/I protein expression levels and corresponding grayscale values. Compared with the Control group: ###p < 0.001; compared with the Model group: *p < 0.05, **p < 0.01, ***p < 0.001; compared with the Gas group: &p < 0.05, &&p < 0.01, &&&p < 0.001; ns, no statistically significant difference, p > 0.05; magnification ×10, scale bar = 200 μm.

To clarify the effect of Gas on autophagic activity, this study compared the effects of Gas on autophagy-related markers under 3-MA treatment conditions. Immunofluorescence results (Figure 3A) showed differences in the intensity and distribution of LC3-related fluorescence signals among different treatment groups. Compared with the Control group, LC3-related fluorescence signals were reduced in the Dox group. Gas treatment enhanced LC3-related fluorescence signals, whereas 3-MA treatment partially attenuated these changes induced by Gas. Western blot results (Figures 3B,C) showed that Gas partially ameliorated Dox-induced abnormalities in p62 accumulation and Beclin-1 and LC3 expression, whereas 3-MA treatment partially reversed the regulatory effects of Gas on these autophagy-related proteins (p < 0.05). These findings suggest that Gas may participate in regulating autophagy-related processes in cardiomyocytes subjected to Dox-induced injury, while the autophagy inhibitor 3-MA partially antagonizes this effect.

### 4.4 The AMPK signaling pathway participates in the cytoprotective effects of gastrodin

**FIGURE 4:**
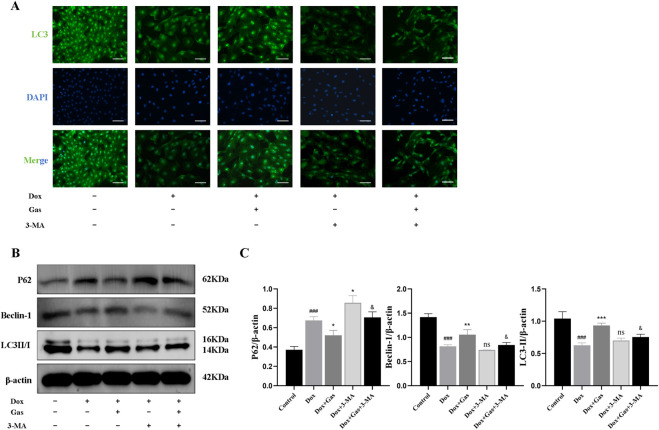
Effects of the AMPK inhibitor CC on the anti-senescence effects of gastrodin. **(A)** Apoptosis analysis by flow cytometry **(B)** Immunofluorescence staining for detection of P16, P21, and P53 expression; **(C)**. Quantitative analysis of immunofluorescence intensity **(D)** Western blot analysis of P16, P21, P53, and γ-H2AX expression **(E)**. Quantitative analysis of P16, P21, P53, and γ-H2AX protein expression levels and corresponding grayscale values. Compared with the Control group: ###p < 0.001; compared with the Model group: **p < 0.01, ***p < 0.001; compared with the Gas group: &p < 0.05, &&&p < 0.001; ns, no statistically significant difference, p > 0.05; magnification ×10, scale bar = 200 μm.

To determine the role of the AMPK signaling pathway in Gas-mediated regulation of cellular senescence and autophagy, the AMPK inhibitor Compound C (CC) was applied. Flow cytometry results (Figure 4A) showed that treatment with the AMPK inhibitor CC alone had no significant effect on the apoptosis rate. However, compared with the Gas group, the apoptosis rate in the Gas + CC co-treatment group was significantly increased, indicating that inhibition of the AMPK pathway antagonized the anti-apoptotic effect of Gas. Regarding senescence-related indicators, immunofluorescence staining (Figures 4B,C) and Western blot analysis (Figures 4D,E) showed that co-treatment with Gas and CC significantly reversed the Gas-induced downregulation of P16, P21, P53, and γ-H2AX expression (p < 0.05), suggesting that Gas suppresses the expression of senescence-associated proteins through activation of the AMPK signaling pathway.

### 4.5 The AMPK inhibitor CC partially reverses the regulatory effects of gastrodin on autophagy

**FIGURE 5:**
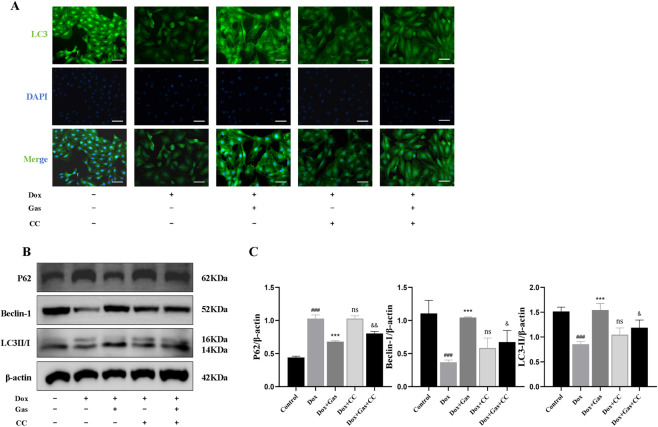
Effects of the AMPK inhibitor CC on gastrodin-mediated regulation of autophagy. **(A)** Immunofluorescence staining for detection of LC3 expression **(B)**. Western blot analysis of P62, Beclin-1, and LC3-II/I expression **(C)**. Quantitative analysis of P62, Beclin-1, and LC3-II/I protein expression levels and corresponding grayscale values. Compared with the Control group: ###p < 0.001; compared with the Model group: *p < 0.05, **p < 0.01, ***p < 0.001; compared with the Gas group: &p < 0.05, &&p < 0.01, &&&p < 0.001; ns, no statistically significant difference, p > 0.05; magnification ×10, scale bar = 200 μm.

To determine whether the AMPK pathway mediates Gas-induced autophagy, we performed Western blotting for LC3-II/I and used immunofluorescence (Figure 5A) as a morphological adjunct. Western blot (Figures 5B,C) showed that CC significantly suppressed the Gas-induced increase in LC3-II expression (p < 0.05). These findings suggest that the protective effect of Gas partially depends on AMPK-mediated regulation of autophagy-related processes.

### 4.6 Gastrodin restores dox-induced signaling imbalance through the AMPK/mTOR/4EBP1 pathway

**FIGURE 6:**
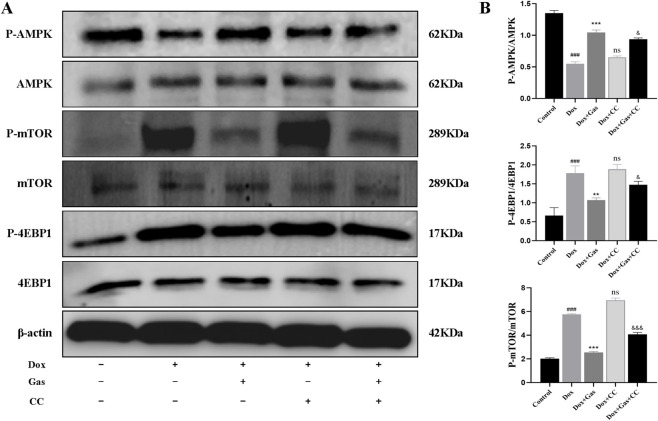
Effects of gastrodin on the AMPK/mTOR/4EBP1 signaling pathway. **(A)** Western blot analysis of the ratios of p-AMPK/AMPK, p-mTOR/mTOR, and p-4EBP1/4EBP1 **(B)**. Quantitative analysis of grayscale values of p-AMPK/AMPK, p-mTOR/mTOR, and p-4EBP1/4EBP1 protein ratios. Compared with the Control group: ###p < 0.001; compared with the Model group: **p < 0.01, ***p < 0.001; compared with the Gas group: &p < 0.05, &&&p < 0.001; ns, no statistically significant difference, p > 0.05; magnification ×10, scale bar = 200 μm.

To elucidate the specific molecular mechanisms underlying the cytoprotective effects of Gas, the phosphorylation levels of key proteins in the AMPK/mTOR/4EBP1 signaling pathway were examined. The results (Figures 6A,B) showed that, compared with the Control group, the phosphorylation level of AMPK (p-AMPK) was significantly decreased in the Dox model group (p < 0.05), whereas the phosphorylation levels of mTOR (p-mTOR) and its downstream target 4EBP1 (p-4EBP1) were significantly increased (p < 0.05). Following Gas treatment, these alterations were reversed, as evidenced by upregulation of p-AMPK expression and downregulation of p-mTOR and p-4EBP1 levels (p < 0.05). However, in the Gas + CC co-treatment group, the regulatory effects of Gas on these phosphorylation events were significantly reversed (p < 0.05). These findings indicate that Gas specifically activates AMPK while inhibiting its downstream mTOR/4EBP1 signaling pathway. Inhibition of AMPK blocks the regulatory effects of Gas, confirming that the AMPK/mTOR/4EBP1 signaling pathway serves as a critical upstream regulatory axis in Gas-mediated autophagy activation and cytoprotection.

## 5 Discussion

Cardiac aging represents one of the key biological foundations driving the increasing burden of age-related cardiovascular events such as heart failure. Current therapeutic strategies mainly focus on controlling risk factors and terminal phenotypes, while interventions targeting the aging mechanism itself remain relatively limited. Therefore, developing strategies capable of blocking or delaying the progression of cardiomyocyte senescence is of practical significance (Childs et al., 2018; Lin et al., 2021).

Gastrodin, the principal active component of *Gastrodia elata*, has been reported to alleviate myocardial injury under stress conditions such as ischemia–reperfusion by promoting autophagic flux (Fu et al., 2018), and to improve mitochondrial function and enhance cell survival in oxidative stress-induced H9c2 models (Cheng et al., 2020). These findings suggest that gastrodin possesses considerable potential as a candidate agent for anti-senescence intervention in cardiomyocytes.

SA-β-gal staining is a classical histochemical marker of cellular senescence and is widely used to identify stress-induced or replicative senescent cells (Dimri et al., 1995). P16 and P21 are cyclin-dependent kinase inhibitors that maintain the retinoblastoma (RB) protein in a hypophosphorylated state and inhibit the G1/S transition, thereby reinforcing and maintaining irreversible cell cycle arrest (González-Gualda et al., 2021). P53 is a central transcription factor in DNA damage responses and stress signaling, and its sustained activation induces downstream effector molecules such as p21, thereby promoting the establishment of the senescence program (Kumari and Jat, 2021). γ-H2A.X is a key DNA damage response marker formed after DNA double-strand breaks; its persistent accumulation indicates sustained DNA damage and DNA damage response (DDR) activation, and is considered an important molecular indicator of genomic instability and DNA damage accumulation during aging (d'Adda di Fagagna, 2008; Siddiqui et al., 2015; da Silva and Schumacher, 2019). In the present study, Dox induced obvious senescence-like changes in H9c2 cells, as shown by an increased proportion of SA-β-gal-positive cells and upregulated expression of P16, P21, P53, and γ-H2A.X, accompanied by elevated apoptosis and decreased cell viability, suggesting that Dox simultaneously activates stress responses associated with DNA damage, cell cycle arrest, and apoptosis. It is noteworthy that although cellular senescence and apoptosis are both important cell fate outcomes after stress, they are not fully equivalent; persistent DNA damage and mitochondrial dysfunction may concurrently drive the establishment of senescence programs and the activation of apoptosis (d'Adda di Fagagna, 2008; Octavia et al., 2012; Childs et al., 2014; Wiley and Campisi, 2021). Previous studies have demonstrated that Dox induces senescence-associated alterations in cardiac tissue (Meléndez et al., 2023), and can activate DDR through induction of DNA damage and γ-H2A.X accumulation (Octavia et al., 2012), further mediating activation of senescence-related pathways such as p53-p21/p16 and disruption of mitochondrial homeostasis, ultimately contributing to anthracycline-induced delayed cardiotoxicity and heart failure (Spallarossa et al., 2009; Foglio et al., 2023; Linders et al., 2024). The present study further demonstrated that Gas dose-dependently ameliorated Dox-induced senescence-associated phenotypes, concurrently reduced apoptosis, and increased cell viability, indicating a comprehensive protective effect against Dox-induced cardiomyocyte injury. Since cellular senescence and apoptosis may share some upstream regulatory mechanisms under persistent stress conditions, the simultaneous improvement of both by Gas may suggest that it acts at more upstream links of stress damage and energy metabolism imbalance. These findings are consistent with previous reports demonstrating cytoprotective effects of Gas in cardiomyocyte stress models. For example, Gas has been shown to improve mitochondrial function and enhance cellular stress resistance in oxidative injury-induced H9c2 models (Cheng et al., 2020). Therefore, the coordinated improvement of senescence markers and apoptosis by Gas may suggest that it can alleviate senescence-associated phenotypes and cellular damage induced by persistent stress by reducing upstream stress burdens such as oxidative stress, DNA damage, and energy metabolism imbalance.

Disruption of autophagic homeostasis has been recognized as a critical mechanism contributing to cardiomyocyte senescence and functional decline (Zheng et al., 2011; Sciarretta et al., 2018). LC3, Beclin-1, and p62 are key autophagy-related markers. The LC3-II level is commonly used to reflect autophagy-related changes, but its increase or decrease alone cannot distinguish between autophagy induction and impaired autophagic degradation (Klionsky et al., 2008). Decreased Beclin-1 expression generally indicates impaired autophagy initiation (Kang et al., 2011). Accumulation of p62 is typically considered an indirect indicator of impaired autophagic degradation, but its expression may also be influenced by transcriptional regulation (Wu et al., 2020). In the present study, Dox treatment led to decreased LC3 and Beclin-1 expression along with p62 accumulation, suggesting the presence of autophagy-related abnormalities, but it is not sufficient to clearly distinguish between inhibition of autophagy initiation or late-stage flux impairment. Notably, previous studies suggest that the effect of Dox on cardiomyocyte autophagy is complex and may be influenced by differences in dose, treatment duration, and detection methods. Li et al. found that Dox can impair autophagic flux by inhibiting lysosomal acidification. The molecular changes observed in this study using 0.1 μM Dox for 24 h may reflect an autophagy-related abnormal state under specific experimental conditions, but are not yet sufficient to unequivocally distinguish between suppressed autophagy initiation and late-stage flux impairment (Li et al., 2016). Meanwhile, Gas partially corrected the abnormalities of these autophagy-related markers and concurrently improved senescence and apoptosis phenotypes. More importantly, when the autophagy inhibitor 3-MA was introduced, the protective effects of Gas on reducing apoptosis and downregulating senescence markers were partially attenuated, and the improvement in LC3, Beclin-1, and p62 expression was also diminished. 3-MA (3-methyladenine) is a classical autophagy inhibitor that blocks autophagosome formation and suppresses autophagy initiation (Wu et al., 2010). These results suggest that autophagy-related processes may be involved in the protective effects mediated by Gas. Previous studies investigating the relationship between Dox and autophagy suggest that Dox-induced autophagic abnormalities are not limited to a single regulatory step. Such alterations may manifest as impaired autophagy initiation or as late-stage defects involving lysosomal function and autophagosome–lysosome fusion, resulting in disrupted autophagic flux (Bartlett et al., 2016; Li et al., 2016; Toda et al., 2023). Additionally, Dox has been reported to suppress basal autophagy through activation of the Akt/mTOR pathway and downregulation of Beclin-1, which is associated with cardiomyocyte apoptosis and structural remodeling (Pizarro et al., 2016; Zhang S. et al., 2023). Therefore, the observed decrease in LC3-II and accumulation of p62 in this study system suggest that autophagy-related clearance processes may be impaired, and the correction of these molecular features by Gas together with partial antagonism by 3-MA jointly suggest that autophagy-related processes may participate in the protective effect of Gas against Dox-induced injury.

Mechanistically, AMPK promotes autophagy-related processes through inhibition of mTORC1 and represents a key adaptive response under conditions of energy stress (Inoki et al., 2003; Gwinn et al., 2008). In recent years, decreased AMPK activity has been linked to cardiac aging, mitochondrial dysfunction, and age-related energy metabolism imbalance, and thus AMPK is also regarded as an important potential intervention target for delaying myocardial aging. In the cardiovascular system, mTORC1 functions as a central signaling hub that coordinates protein synthesis and metabolic adaptation while interacting closely with autophagy regulation. Thus, AMPK-mediated inhibition of mTORC1 can be interpreted as a protective stress adaptation mechanism (Sciarretta et al., 2018). 4EBP1 is a classic downstream effector of mTORC1, and changes in its phosphorylation level can reflect the activation state of mTORC1 (Gingras et al., 2001). Furthermore, the AMPK inhibitor Compound C (CC) attenuated the protective effects of Gas on apoptosis rate, senescence markers, and autophagy-related indices at the phenotypic level, collectively supporting that the AMPK/mTOR/4EBP1 pathway may be involved in Gas-mediated protection. Consistently, pathway analysis showed that Dox reduced the p-AMPK/AMPK ratio while increasing the p-mTOR/mTOR and p-4EBP1/4EBP1 ratios. Gas treatment partially restored AMPK phosphorylation and suppressed excessive phosphorylation of mTOR and 4EBP1, whereas the addition of CC diminished these improvements to varying degrees. Taken together, the pharmacological antagonism results and the direction of pathway activation revealed by phosphorylation changes jointly suggest that the protective effect of Gas may be related to regulation of this pathway.

Despite these findings, several limitations should be acknowledged. First, the assessment of autophagic status in this study relied primarily on static indicators such as LC3-II/I, Beclin-1, and p62, which are insufficient to rigorously distinguish enhanced autophagy initiation from late-stage blockage of autophagic flux leading to autophagosome accumulation. Future studies will incorporate lysosomal inhibitors to conduct LC3/p62 accumulation assays. Second, functional validation of AMPK mainly depended on Compound C, which may have potential off-target effects. Therefore, subsequent studies will employ AMPK knockdown strategies to further substantiate the critical role of AMPK in Gas-mediated protective effects. In addition, this study mainly established a Dox-induced senescence-like model in H9c2 cells, and still lacks validation in primary cardiomyocytes and *in vivo* natural aging models, which will be further investigated in future studies.

## 6 Conclusion

**FIGURE 7:**
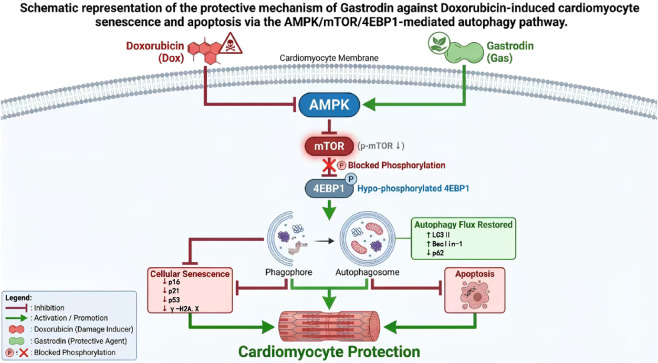
Schematic diagram of the potential mechanism by which gastrodin alleviates Dox-induced cardiomyocyte senescence. Dox treatment inhibits AMPK activity and activates the mTOR/4EBP1 signaling pathway, accompanied by abnormalities in autophagy-related markers, elevated expression of senescence markers, and increased apoptosis. Gas intervention partially restores AMPK activity and suppresses excessive phosphorylation of mTOR/4EBP1, while ameliorating abnormalities in autophagy-related markers and attenuating senescence and apoptosis phenotypes, ultimately mitigating Dox-induced cardiomyocyte injury.

This study demonstrates that Gas significantly alleviates Dox-induced cardiomyocyte senescence and apoptosis, accompanied by improvement of autophagy-related marker abnormalities. Mechanistically, this effect is associated with activation of AMPK and inhibition of the mTOR/4EBP1 signaling axis, and both AMPK inhibitor and autophagy inhibitor partially attenuated the protective effects of Gas, suggesting that the AMPK/mTOR/4EBP1 pathway and autophagy-related processes may be involved in its mechanism of action (Figure 7). In summary, Gas may regulate autophagy-related processes through the AMPK/mTOR/4EBP1 signaling pathway, thereby reducing Dox-induced cardiomyocyte senescence and related injury, providing new experimental evidence for natural active ingredient intervention against cardiac aging.

## Data Availability

The original contributions presented in the study are included in the article/supplementary material, further inquiries can be directed to the corresponding authors.
